# Supplementary material for: Mycobacterium tuberculosis, Mycobacterium kansasii and Rhodococcus equi induce macrophage necroptosis in the presence of a caspase inhibitor acting on a non-canonical target(s)
Source: bioRxiv. 2025 Sep 2:2025.09.02.673674. Preprint. [Version 1] doi: 10.1101/2025.09.02.673674 (PMC12424694; doi:10.1101/2025.09.02.673674)

bioRxiv preprint doi: https://doi.org/10.1101/2025.09.02.673674; this version posted September 2, 2025. The copyright holder for this preprint (which was not certified by peer review) is the author/funder, who has granted bioRxiv a license to display the preprint in perpetuity. It is made available under aCC-BY 4.0 International license.

Figure S1. z-VAD sensitizes Mtb-infected BMDMs to necroptosis, which relies on host RIPK3 and MLKL, related to Figure 1. For infection experiment, BMDMs were pretreated with compounds at half of the indicated concentration for 2 h before infection of Mtb and then indicated concentration (20 μM except that GSK'872 is used at 3 μM) after washing Mtb away till harvest. (A and B) Effects of various caspase inhibitors on Mtb-infected macrophages. WT BMDMs were pretreated with indicated compounds and cell viability was measured on 1 d.p.i. (C) z-VAD, Nec-1 and GSK'872 are not cytotoxic without Mtb infection. WT BMDMs were treated with indicated compounds for 24 h and then assayed for cell viability. (D) Mtb + z-VAD induced RIPK1 and MLKL phosphorylation in a time-dependent manner. WT BMDMs were pretreated with indicated compounds 2 h before being infected with Mtb at an MOI of 10. Cell lysates were harvested at indicated time and subjected to immunoblots. (E) MLKL is essential for Mtb + z-VAD-triggered necroptosis. BMDMs were pretreated with indicated compounds 2 h before being infected with Mtb at an MOI of 15. Cells were assayed 1 d.p.i. (f and g) Mutation of *Ripk3* or loss of *Mlkl* in BMDMs renders cells resistant to TSZ-triggered necroptosis. BMDMs were treated with indicated compounds for 4 h and then assayed for cell viability by measuring ATP level. (h) The viability curve of WT BMDMs in response to different necroptotic stimulus. Cell viability was measured by ATP level on 3, 6 and 24 h after treatment. (i) Mtb + z-VAD did not induce classical RIPK3 phosphorylation. Cells were treated, infected and assayed as in (d). TS and TSZ treatment lasted for 2 h. T, TNFα, 10 ng/mL; S, Smac-mimetic birinapant, 10 μM; Z, z-VAD, 20 μM; Q, Q-VD-OPH, 20 μM; G, GSK'872, 3 μM; N, Nec-1, 20 μM. Data shown as means ± SD of three technical replicates (a-c and e-h) and representative of two independent experiments.

Figure S2. Mtb-related necroptosis depends on TLR2-TNF signaling as well as type I IFN signaling, related to Figure 2. (A-B, D-I) Effects of various gene deficiencies on Mtb-related macrophage necroptosis based on cell viability assay in the presence of Mtb + z-VAD. BMDMs from WT and indicated knockout mice were pretreated compounds with at 10 μM for 2 h before infection of Mtb at an MOI of 20 and then 20 μM after washing Mtb away till harvest. Cell viability was assayed 1 d.p.i. by

bioRxiv preprint doi: https://doi.org/10.1101/2025.09.02.673674; this version posted September 2, 2025. The copyright holder for this preprint (which was not certified by peer review) is the author/funder, who has granted bioRxiv a license to display the preprint in perpetuity. It is made available under aCC-BY 4.0 International license.

measuring ATP level. Data are means ± SD of three technical replicates. Data are summarized in Figure 2A. (C) WT and *Tlr2*$^{-/-}$ BMDMs were infected with Mtb and culture supernatant was harvested 24 h.p.i. and measured by TNFα ELISA. N.D., not detected.

Figure S3. Mtb-induced production of TNFα and type I IFNs are important for necroptosis triggered by Mtb + z-VAD, related to Figure 2. (A) TNFα plus z-VAD does not elicit necroptosis. WT BMDMs were treated with indicated compounds for 31 h and cell viability was analyzed by measuring ATP level. (B) The combination TNFα and type I IFN does not replace Mtb infection in inducing necroptosis. Cells are treated with indicated compounds and cytokine for 25 h and then assayed as in (A). T, TNFα, 10 ng/mL; S, Smac-mimetic birinapant, 10 μM; Z, z-VAD, 20 μM; IFN-α, 1,000 U/ml; IFN-β: 100, 500, and 1,000 U/ml.

Figure S4. *R.equi* activates type I IFN signaling and induces RIPK3 and MLKL-dependent cell death in the presence of z-VAD, related to Figure 4. (A) qPCR analysis on *R.equi*-infected BMDMs at 4 h.p.i (left) and 8 h.p.i (right). Total RNA was extracted and used for cDNA reverse transcription before qPCR analysis. (B) J774.1 cells were pretreated at half of the indicated concentration for 2 h before *R. equi* 33701-strain infection and then indicated concentration till measuring ATP level 24 h.p.i. KO cells are generated by CRISPR-Cas9–mediated targeting independently using gRNA1 or 2 for both *Ripk3* and *Mlkl*. D, DMSO; Z, z-VAD, 20 μM; N, Nec-1, 20 μM. Data shown as means ± SD of three technical replicates and representative of two independent experiments. ns, not significant; ****, p< 0.0001 (two-tailed unpaired Student's *t* test).

Figure S5. Medium dose of Mtb infection in *Sp140*$^{-/-}$ mice results in slow TB progression, related to Figure 5. *Sp140*$^{-/-}$ and *Sp140*$^{-/-}$*Mlkl*$^{-/-}$ mice were infected with 400-500 CFU of Mtb on day 0. For (B-F), mice were euthanized on day 58. (A) Weight changes of Mtb-infected mice. (B) Mtb burdens in lungs (left), spleens (middle) and livers (right) of mice. (C) The ratio of lung (up) and spleen (down) weight to whole body weight in Mtb-infected mice. (D) Representative HE staining of lung sections of Mtb-infected mice. (E) Pathology score of lung sections of Mtb-infected mice. n=11 for

bioRxiv preprint doi: https://doi.org/10.1101/2025.09.02.673674; this version posted September 2, 2025. The copyright holder for this preprint (which was not certified by peer review) is the author/funder, who has granted bioRxiv a license to display the preprint in perpetuity. It is made available under aCC-BY 4.0 International license.

Sp140$^{-/}$ mice and n=7 for *Sp140*$^{-/-}$*Mlkl*$^{-/-}$ mice**.** Data are means ± SEM (A-C and E). ns, not significant; *, $p < 0.05$.(two-tailed unpaired Student's *t* test).

bioRxiv preprint doi: https://doi.org/10.1101/2025.09.02.673674; this version posted September 2, 2025. The copyright holder for this preprint (which was not certified by peer review) is the author/funder, who has granted bioRxiv a license to display the preprint in perpetuity. It is made available under aCC-BY 4.0 International license.

Figure S1

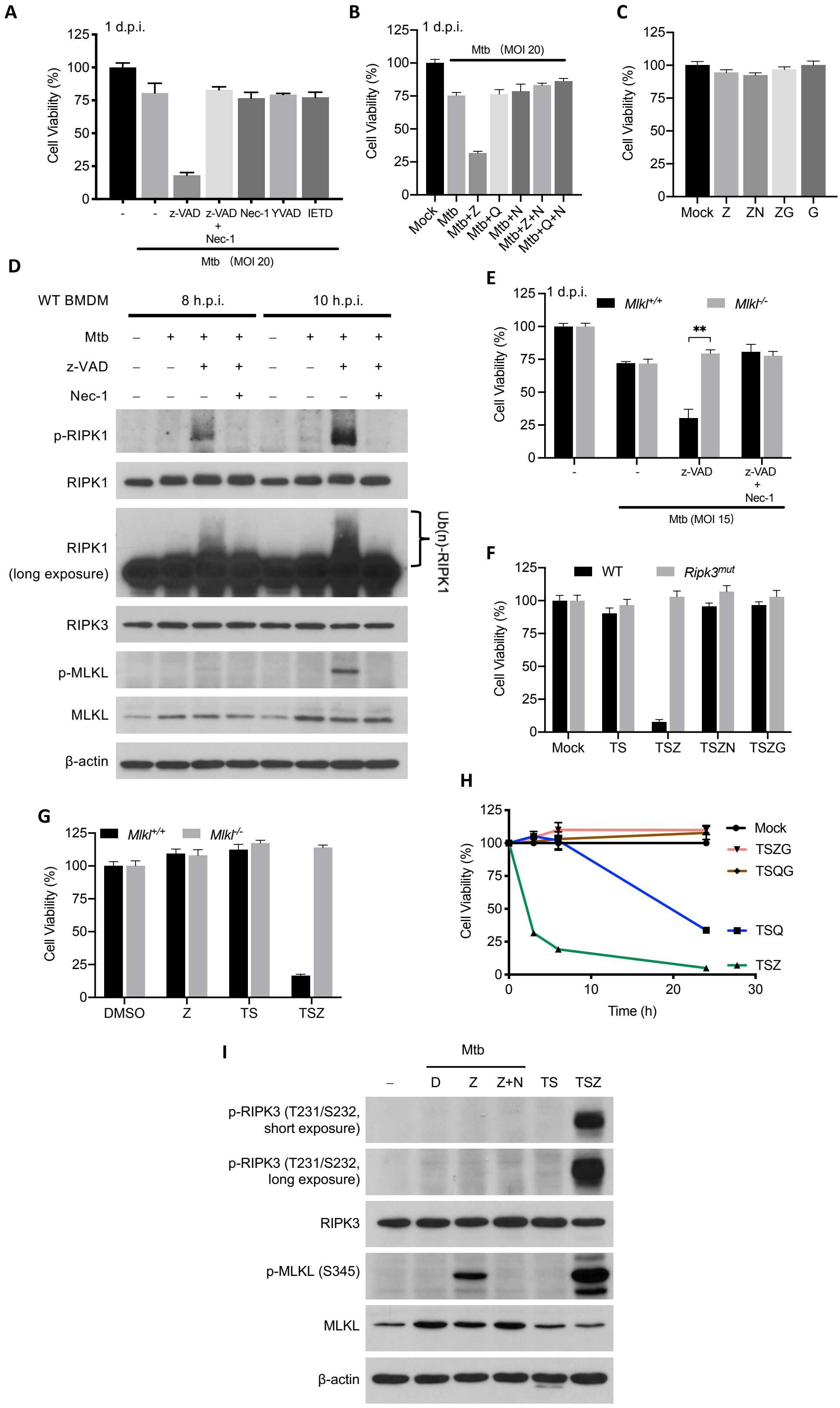

bioRxiv preprint doi: https://doi.org/10.1101/2025.09.02.673674; this version posted September 2, 2025. The copyright holder for this preprint (which was not certified by peer review) is the author/funder, who has granted bioRxiv a license to display the preprint in perpetuity. It is made available under aCC-BY 4.0 International license.

Figure S2

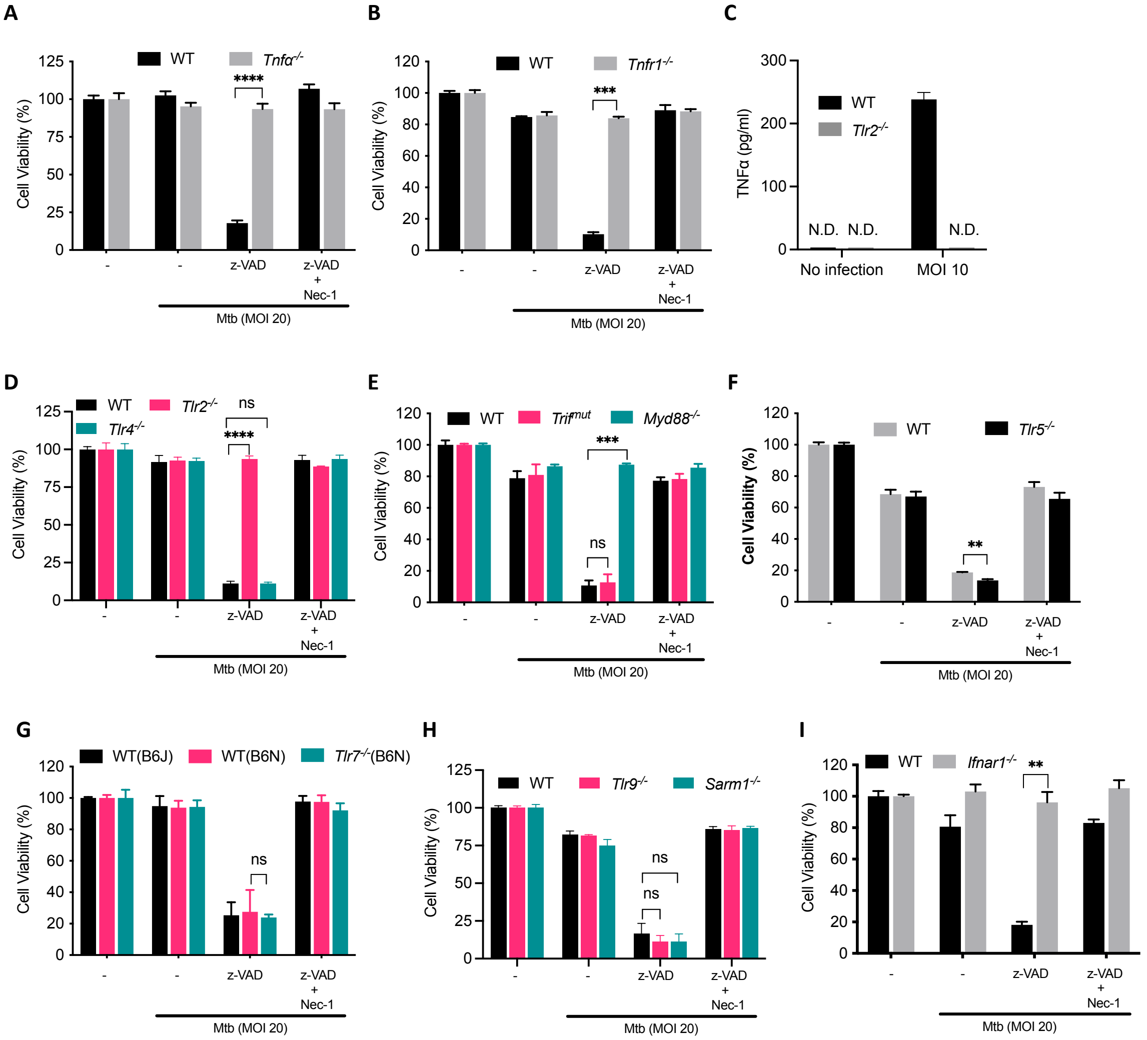

bioRxiv preprint doi: https://doi.org/10.1101/2025.09.02.673674; this version posted September 2, 2025. The copyright holder for this preprint (which was not certified by peer review) is the author/funder, who has granted bioRxiv a license to display the preprint in perpetuity. It is made available under aCC-BY 4.0 International license.

Figure S3

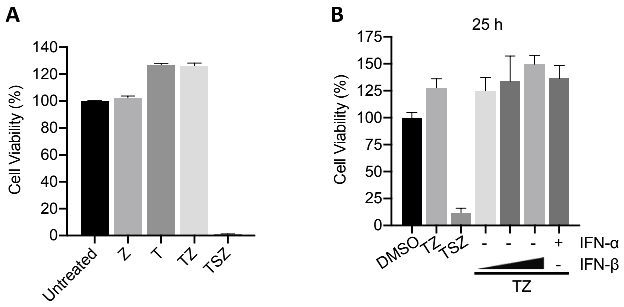

bioRxiv preprint doi: https://doi.org/10.1101/2025.09.02.673674; this version posted September 2, 2025. The copyright holder for this preprint (which was not certified by peer review) is the author/funder, who has granted bioRxiv a license to display the preprint in perpetuity. It is made available under aCC-BY 4.0 International license.

Figure S4

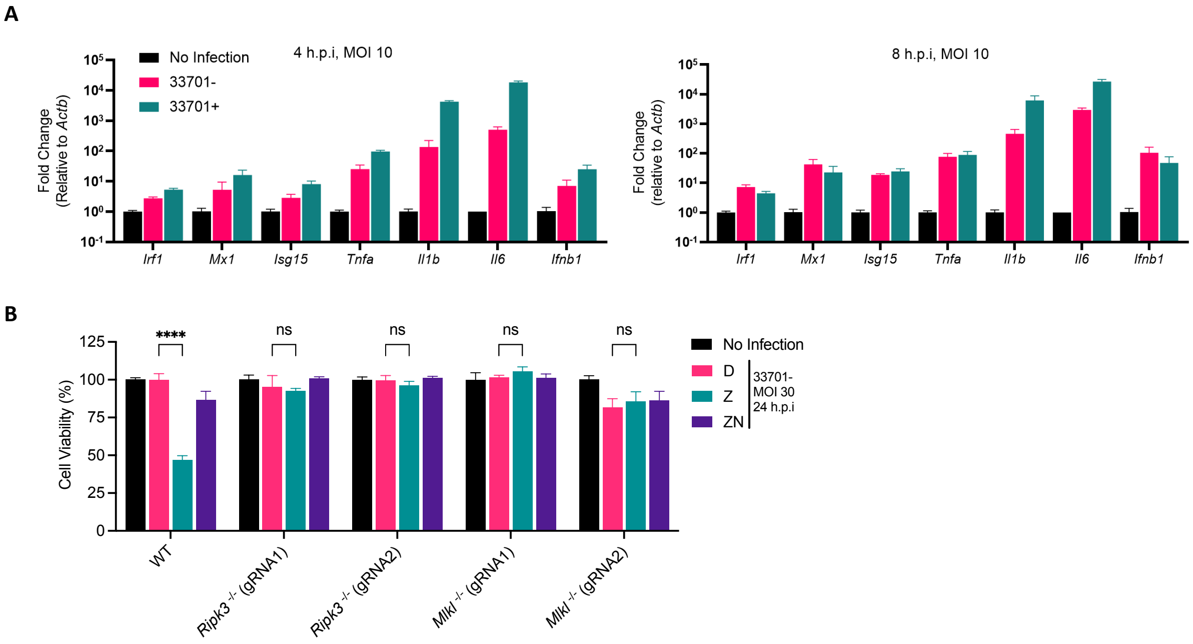

bioRxiv preprint doi: https://doi.org/10.1101/2025.09.02.673674; this version posted September 2, 2025. The copyright holder for this preprint (which was not certified by peer review) is the author/funder, who has granted bioRxiv a license to display the preprint in perpetuity. It is made available under aCC-BY 4.0 International license.

Figure S5

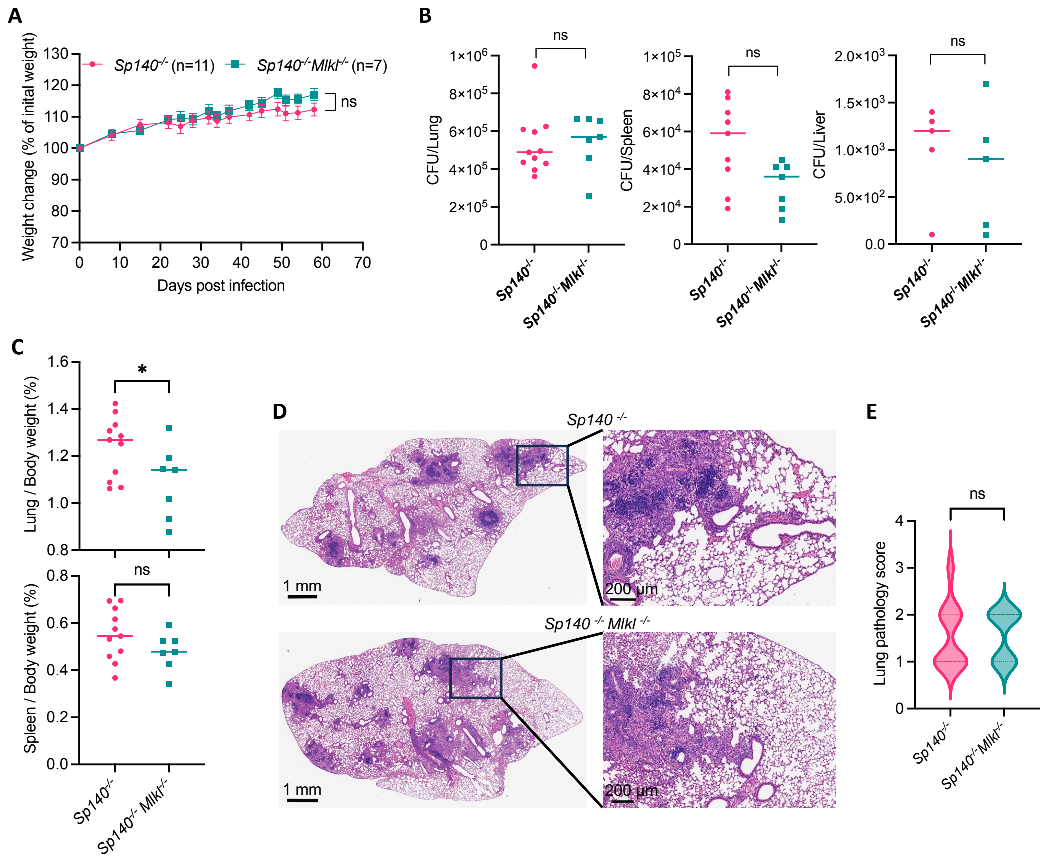

Supplement: Supplement 1 [file NIHPP2025.09.02.673674v1-supplement-1.pdf]
